# Acute pulmonary embolism following dual-chamber pacemaker implantation: a case report

**DOI:** 10.3389/fcvm.2025.1587204

**Published:** 2025-10-30

**Authors:** Zhizhou Song, Yanhua Zhang, Yanan Wang, Lijuan Hao, Ying Ma, Qiang Liu, Qi Wu, Yuehong Huo

**Affiliations:** ^1^ Department of Cardiology, The Third People’s Hospital of Datong, Datong, Shanxi, China; ^2^ Department of Cardiology, Shanxi Province Cardiovascular Disease Hospital, Taiyuan, Shanxi, China; ^3^ Department of Cardiology, Shanxi Province Guoyao Tongmei General Hospital, Datong, Shanxi, China; ^4^ Department of Rheumatology, The Fifth People’s Hospital of Datong, Datong, Shanxi, China

**Keywords:** acute pulmonary embolism, dual-chamber pacemaker, intravenous thrombolysis, pocket hematoma, cardiogenic shock

## Abstract

Acute pulmonary embolism (APE), a critical complication following permanent pacemaker implantation, presents profound therapeutic challenges when occurring during the early postoperative phase. We report a 73-year-old female who developed high-risk APE with cardiogenic shock 47 h after dual-chamber pacemaker implantation via the right subclavian vein. The immobilization of the affected upper limb and bed rest, along with endothelial injury during the implantation process, can trigger an inflammatory response and activate the coagulation cascade, ultimately leading to a pro-coagulant state, which may subsequently induce deep vein thrombosis in the lower extremities and subsequent bilateral pulmonary embolism. Following the 2019 ESC guidelines for managing high-risk APE, prompt intravenous thrombolysis with alteplase (50 mg) stabilized hemodynamics. However, this intervention caused pacemaker pocket hemorrhage. Strategic intermittent elastic compression bandaging mitigated hematoma progression without compromising wound healing. Anticoagulation with warfarin (INR 2–3) and serial imaging confirmed resolution of thromboembolic burden and right atrial remodeling. This case underscores the delicate balance between life-saving reperfusion and device-related complications in pacemaker recipients, advocating for tailored hemostatic strategies in high-risk cohorts.

## Introduction

Acute pulmonary embolism (APE), the most severe manifestation of venous thromboembolism (VTE), ranks as the third leading cause of cardiovascular mortality, surpassed only by coronary artery disease and stroke (1). High-risk APE carries a 30-day mortality rate of 22% (2), underscoring the critical need for timely intervention to optimize survival and clinical outcomes. While rare, APE constitutes a life-threatening complication following permanent pacemaker implantation, particularly when occurring during the vulnerable postoperative period prior to wound and device pocket healing.

The management of APE in this context presents a formidable therapeutic paradox. First-line therapy for high-risk APE requires immediate reperfusion, typically via systemic thrombolysis, to ease right ventricular strain and restore hemodynamic stability. However, thrombolytic agents and subsequent anticoagulation markedly elevate the risk of pocket hematoma and infection in pacemaker recipients, potentially necessitating device explantation or electrode extraction. Such interventions impose significant financial burdens and mortality risks, rendering clinical decision-making exceptionally complex.

Pacemaker implantation inherently predisposes patients to thromboembolic events through Virchow's triad: procedural endothelial injury, postoperative immobilization, and hypercoagulability. These factors synergistically increase susceptibility to lower extremity deep vein thrombosis (DVT) and subsequent APE. Notably, the early postoperative phase-a critical window for wound healing-demands meticulous balancing of thromboprophylaxis against hemorrhagic complications. Current guidelines emphasize minimizing immobilization duration and optimizing surgical precision, particularly in elderly populations with heightened thromboembolic vulnerability.

This case report highlights the intricate challenges of managing high-risk APE in a pacemaker recipient, where thrombolysis-induced hematoma threatened device integrity. By integrating evidence-based reperfusion strategies with innovative hemostatic techniques, we navigated the precarious balance between life-saving intervention and procedural success, offering insights into tailored management protocols for this high-stakes clinical scenario.

## Case presentation

**Figure 1:**
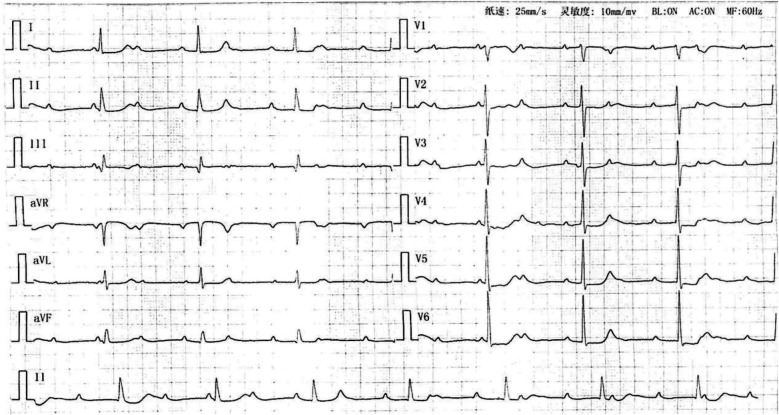
Electrocardiogram showing sinus rhythm with third-degree atrioventricular block.

A 73-year-old woman presented on July 3, 2023, with a two-month history of exertional dyspnea, chest tightness, palpitations, and fatigue, exacerbated over four days. Progressive decline in functional capacity culminated in dyspnea after ambulating 50 meters. Admission electrocardiogram revealed third-degree atrioventricular block with junctional escape rhythm (Figure 1). Medical history included hypertension (maximum 180/110 mmHg, controlled with nifedipine 20 mg twice daily) and newly diagnosed hyperglycemia (fasting glucose 8–10 mmol/L, managed via diet). Initial D-dimer measured 190 μg/L (reference: 0–550 μg/L). Echocardiography demonstrated biatrial enlargement (right atrium 41 mm, left atrium 37 mm), tricuspid regurgitation, and preserved left ventricular ejection fraction (64%).

**Figure 2:**
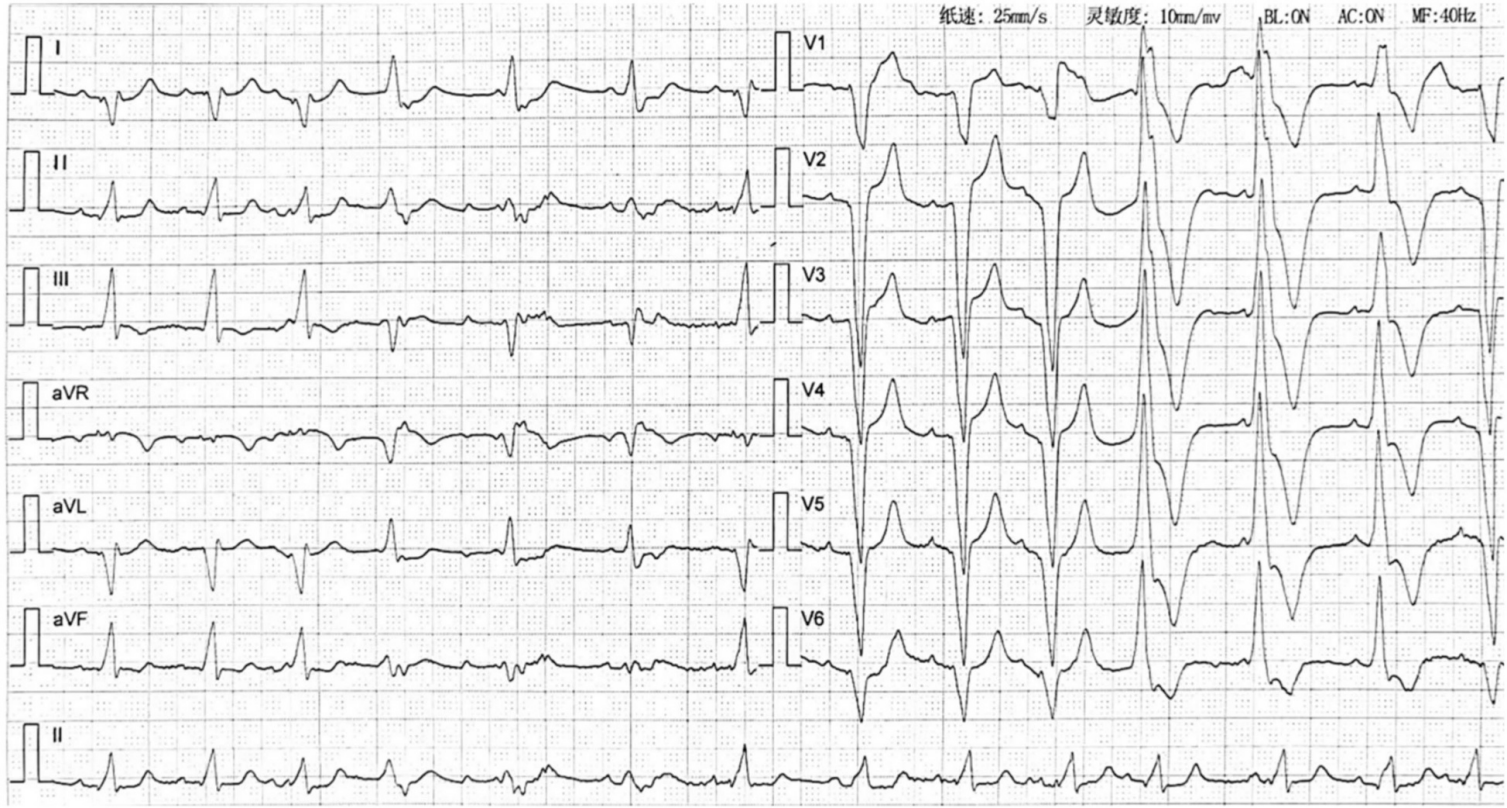
Electrocardiogram after pulmonary embolism (2023-09-19) showing accelerated idioventricular rhythm with atrioventricular dissociation.

**Figure 3:**
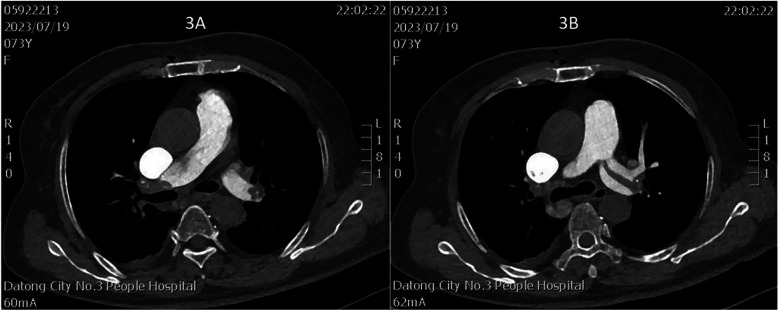
**(A)** Thrombus in the right main pulmonary artery; **(B)** thrombus in the left main pulmonary artery.

**Figure 4:**
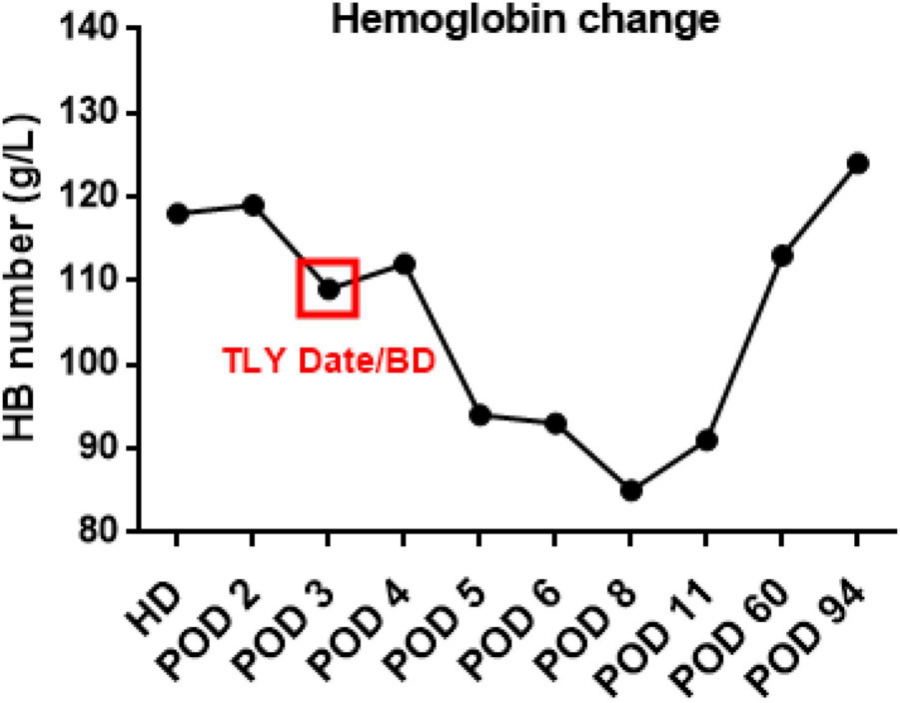
Trends in hemoglobin levels over time. (HD, hospitalization day; POD, postoperative day; TLY, thrombolysis; BD, bleeding day).

**Figure 5:**
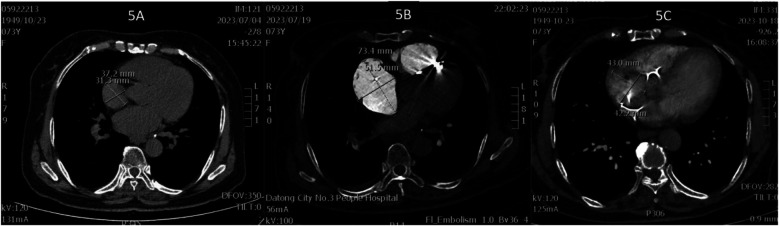
**(A)** Right atrial diameter upon admission; **(B)** right atrial diameter 2 h after pulmonary embolism; **(C)** right atrial diameter 3 months after pulmonary embolism.

**Figure 6:**
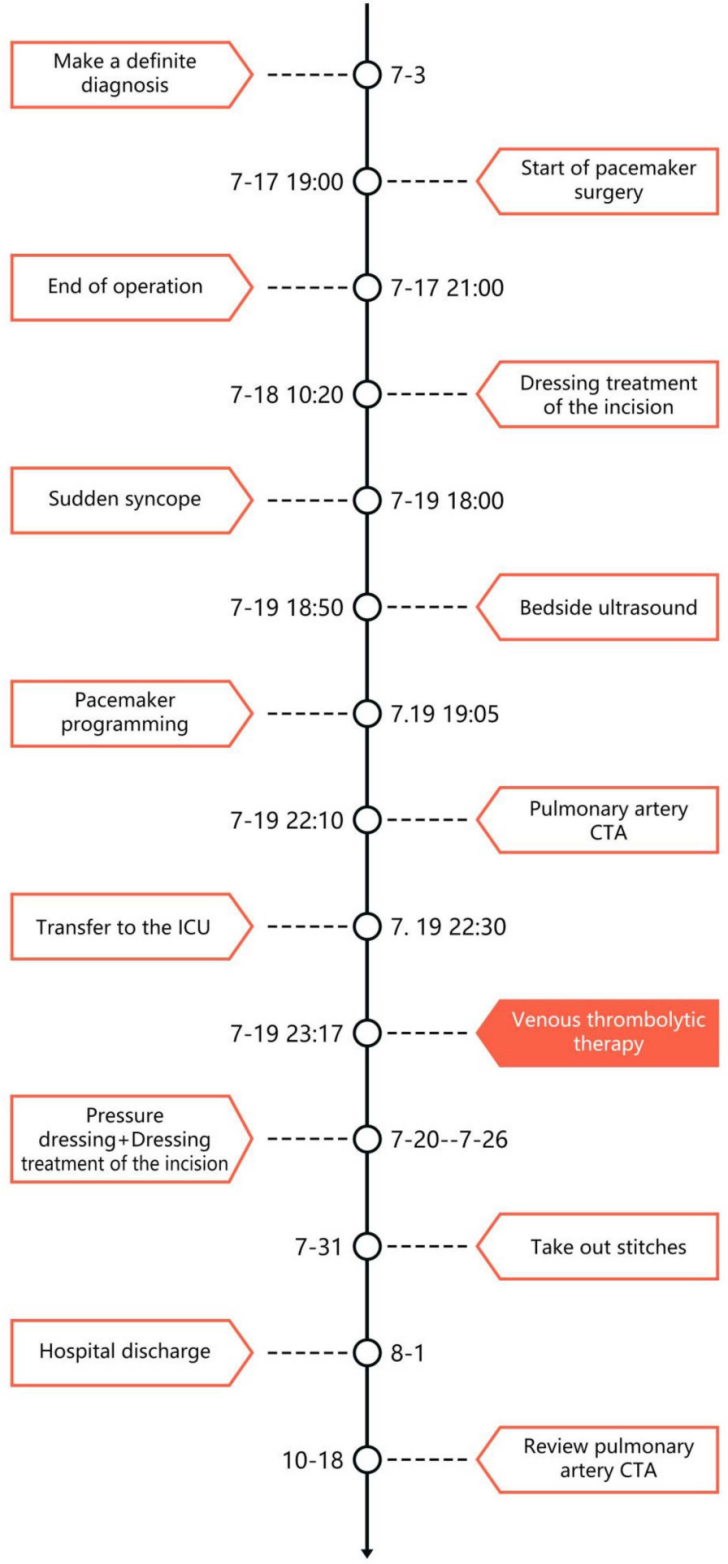
Timeline.

On July 17, a dual-chamber pacemaker (St. Jude Medical PM2172) was implanted via the right subclavian vein under standard parameters: atrial impedance 652 Ω, threshold 1.0 V, P-wave amplitude 10.6 mV; ventricular impedance 768 Ω, threshold 0.5 V, R-wave amplitude 13.6 mV. Strict immobilization of the operative-side upper limb was required within 24 h after surgery. During this period, patients were absolutely prohibited from raising the affected arm above shoulder level and from using the operated-side arm to support themselves when getting out of bed. Additionally, patients were instructed to avoid lying on the surgical side and were encouraged to begin ambulation starting 3 h after surgery. With the day of surgery designated as postoperative day 1 (POD1). By POD2, the incision site remained dry and showed no signs of pocket swelling. At 47 h post-implantation, the patient experienced syncope during ambulation, accompanied by hypotension (76/50 mmHg), tachycardia (110 bpm), tachypnea (30–40/min), and hypoxemia (SpO2 82%). D-dimer surged to 1,617 μg/L. Electrocardiographic findings included VAT (Ventricular pacing, Atrial sensing, and Triggered response) pacing mode with accelerated idioventricular rhythm and atrioventricular dissociation (Figure 2). Device interrogation revealed atrial sensing failure and elevated thresholds, prompting transition to VVI pacing (Table 1). Bedside echocardiography at 18:50 showed: right ventricular enlargement (30 mm), paradoxical motion of the interventricular septum, and moderate pulmonary hypertension (tricuspid peak systolic pressure gradient >60 mmHg). Based on the patient's symptoms, signs, and relevant examinations, the possibility of acute pulmonary embolism was considered high, and immediate treatments were administered, including oxygen inhalation, approximately 800 ml of fluid replacement, and norepinephrine for blood pressure elevation. After the vital signs became slightly stable (blood pressure: 98/60 mmHg, heart rate: 100 beats per minute, respiratory rate: 25 breaths per minute, blood oxygen saturation: 92%), a pulmonary computed tomography angiography (CTA) performed at 22:10 confirmed bilateral pulmonary artery emboli (Figure 3). The patient was transferred to the ICU at 22:30. Alteplase (50 mg) thrombolysis initiated at 23:17 (POD3) yielded rapid symptomatic improvement (SpO2 98% on 5 L/min O2 within two hours). Subsequent low-molecular-weight heparin (5,000 U subcutaneously every 12 h) was administered. By POD3, fresh hemorrhage and pocket distension necessitated intermittent compressive dressing (10 h compression/2 h release for four days, transitioning to 6 h cycles thereafter). On the second day after thrombolysis (POD4), tumor markers were measured in the patient, and the results showed that alpha-fetoprotein (AFP), carcinoembryonic antigen (CEA), carbohydrate antigen 125 (CA125), carbohydrate antigen 15-3 (CA15-3), and squamous cell carcinoma antigen (SCCA) were all within the normal range. The pocket ecchymosis measured approximately 20 cm × 15 cm, but no skin necrosis occurred. No hemorrhage was observed in other organs. Changes in hemoglobin levels are shown in Figure 4. Despite periprocedural ecchymosis, lower extremity ultrasonography on POD8 revealed bilateral calf muscular venous thrombosis. Warfarin anticoagulation commenced on POD9, maintaining an INR of 2–3. The incision healed without complication by POD12. A follow-up echocardiogram on POD8 revealed only a small amount of tricuspid regurgitation. Follow-up evaluations at 28 days and three months post-discharge demonstrated stable pacemaker function (Table 1), resolved pulmonary emboli, and reduced right atrial dimensions (73.4 × 51.5 mm to 43.0 × 42.2 mm; Figure 5). The key time points of the patient are shown in Figure 6.

**Table 1:** Testing results of pacemaker parameters.

Test date	Testing time period	Atrial parameters	Ventricular parameters
		Atrial sensing (mV)	Atrial threshold (V)
July 18	Immediately Post-Operation	3.6	1.0
July 19	1 h after Pulmonary Embolism	0.6	3.0
July 22	4 days after Pulmonary Embolism	2.2	1.2
July 31	13 days after Pulmonary Embolism	2.5	0.6
August 15	28 days after Pulmonary Embolism	2.8	0.6
October 18	3 months after Pulmonary Embolism	3.0	0.6

## Discussion

In contemporary clinical practice, the use of pacemakers and implantable cardioverter-defibrillators (ICDs) has become increasingly widespread. Their use may lead to complications, including dislodgement of the pacemaker lead, infection, and venous thrombosis or occlusion after implantation (3). Studies show that the incidence of upper extremity deep vein thrombosis (UEDVT) associated with cardiac implantable devices ranges from 0.5% to 30% (4–6). Currently, it is believed that the mechanism of UEDVT related to cardiac implantable devices involves the lead placed via the vein acting as an intravascular foreign body, which triggers turbulent venous blood flow, leading to platelet aggregation and thrombosis (7). Additionally, endothelial damage during the implantation process can provoke an inflammatory response and activate the coagulation cascade, ultimately resulting in a pro-coagulant state (8, 9). There is also the rare occurrence of post-cardiac injury syndrome leading to pericarditis (10). Furthermore, undergoing cardiac implantable electronic device (CIED) surgery presents not only a physical challenge but also a psychological trial. The entire process, from diagnosis to implantation and adapting to the new device, may induce anxiety, and the continuous interaction between daily activities and device functionality complicates the situation further (11).

APE, though a rare yet consequential complication following permanent pacemaker implantation, exemplifies the intricate interplay between therapeutic intervention and procedural risks. In this reported case, the patient underwent dual-chamber pacemaker placement via the right subclavian vein with 24 h postoperative immobilization. Consistent with Virchow's triad, endothelial injury from venous access and immobilization-induced venous stasis precipitated lower extremity DVT, culminating in APE. As shown in Figure 2, the patient's cardiac rhythm transitioned from sinus rhythm with left bundle branch block (LBBB) to accelerated ventricular tachycardia (AVT) with right bundle branch block (RBBB). This rhythm change is attributed to the presence of thrombi in both the left and right main pulmonary arteries. The core mechanism by which pulmonary embolism induces ventricular tachycardia (VT) is that multiple pathophysiological factors collectively lead to extreme instability of myocardial electrical activity. Specifically, these factors include: acute right ventricular pressure overload and stretch-related mechanical stimulation, acute myocardial ischemia/hypoxia and reperfusion injury, catecholamine storm triggered by neurohumoral activation, acidosis and electrolyte disturbances, as well as the Bezold-Jarisch reflex. These factors interact with each other, jointly disrupting the normal electrophysiological balance of the myocardium and ultimately inducing VT.

The case was classified as high-risk according to the 2019 ESC guidelines for acute PE management (12). Immediate reperfusion, preferably via intravenous thrombolysis, was essential. It aimed to restore pulmonary perfusion, optimize ventilation-perfusion ratios, reduce right ventricular afterload and thus stabilize hemodynamics (13). 5, Domestic studies have shown that the efficacy of continuous intravenous infusion of low-dose rt-PA (50 mg) for 2 h is comparable to that of the dose recommended by the U.S. Food and Drug Administration (FDA, 100 mg), while its safety is superior. Particularly, the incidence of bleeding events is significantly reduced in patients with a body weight of <65 kg (14). The patient reported in our case had a body weight of 60 kg; meanwhile, considering that it was the third day after pacemaker implantation and the risk of pocket bleeding was high, we chose the dose of 50 mg.

However, thrombolytic therapy during the early postoperative phase, compounded by subsequent anticoagulation, exponentially elevates the risk of pacemaker pocket hemorrhage and hematoma formation. Literature indicates a 2%–20% incidence of pocket hematoma in anticoagulated pacemaker recipients (15), with such complications correlating strongly with postoperative infection and impaired wound healing (16–18). Notably, 88% of pocket infections require complete device removal, and 57.7% need reimplantation (19). Research has also shown that if a patient develops an infection in their pacemaker pocket, more than just antimicrobial treatment is needed. In fact, the entire device and its transvenous leads must be removed through a procedure called transvenous lead extraction (TLE). This not only brings a heavy financial burden, with estimated additional hospitalization costs of around $50,000, but also increases the risk of in-hospital death (20, 21). The long-term mortality rate of patients is also significantly increased (22).

This case involves a 73-year-old woman who developed APE with cardiogenic shock 47 h after implantation. Managing it highlights this clinical dilemma. Intravenous alteplase (50 mg) administration achieved rapid symptom resolution within two hours, albeit precipitating pocket hemorrhage and swelling. Intermittent elastic compression bandaging was used (10 h cycles for four days, then transitioning to 6 h intervals). This method effectively mitigated hematoma expansion without causing skin necrosis, which aligns with reported strategies for controlling post-implantation hemorrhage (23). Warfarin was initiated on day seven, with the dosage titrated to maintain an INR of 2–3. Follow-up imaging at three months showed resolved DVT, pulmonary emboli, and right atrial remodeling (from 73.4 × 51.5 mm to 43.0 × 42.2 mm; see Figure 5). These findings underscore the viability of this approach.

Currently, with advancements in device miniaturization, communication, and battery life technology, leadless pacemakers (LPMs) have emerged as a new star in the treatment of bradyarrhythmias. They aim to reduce complications associated with traditional pacemaker leads and pockets. LPMs are approximately one-tenth the size of traditional pacemakers, have a long battery life, enable quick postoperative recovery, and avoid lead and pocket-related complications. Additionally, they feature advanced functions such as adaptive frequency and automatic threshold management, are compatible with magnetic resonance imaging, and have a short training period, among many other advantages (24). The leadless dual-chamber pacemaker Micra AV (which senses the atrium before pacing the ventricle to maintain normal atrioventricular conduction order) has been released on the market. The Micra AV achieves atrioventricular synchronization through mechanical sensing principles (22, 25). However, complications such as pericardial effusion, cardiac perforation, vascular-related complications (arteriovenous fistula, pseudoaneurysm), and device displacement can still occur during the implantation of LPM. The patient reported in our case chose a traditional dual-chamber pacemaker due to economic reasons. Although guidelines from the American Society of Hematology (ASH), American College of Chest Physicians (ACCP), and European Society of Cardiology (ESC) recommend the use of direct oral anticoagulants (DOACs) such as rivaroxaban for the treatment of pulmonary embolism, rather than vitamin K antagonists (VKAs). We took into account that if rivaroxaban were used for anticoagulation, in the event of unpredictable major bleeding, it would be difficult to obtain the specific antagonist Andexanet alfa which reverses the anticoagulant effect of rivaroxaban (and apixaban). This is because the drug is currently hardly available in the Chinese market.

Current evidence remains sparse regarding optimal reperfusion strategies for high-risk APE in pacemaker recipients and hematoma mitigation protocols. Our intervention-thrombolysis combined with staged mechanical compression-not only salvaged the patient's life but also circumvented device extraction or reimplantation. While thromboprophylaxis and vigilant APE recognition remain pivotal in reducing mortality, standardized protocols for post-thrombolytic hematoma management await large-scale validation. This case highlights the necessity for individualized risk-benefit calculus and hemostatic techniques in balancing life-saving reperfusion with device-related complications.

## Data Availability

The original contributions presented in the study are included in the article/Supplementary Material, further inquiries can be directed to the corresponding author.
